# A Nonlinear Double Model for Multisensor-Integrated Navigation Using the Federated EKF Algorithm for Small UAVs

**DOI:** 10.3390/s20102974

**Published:** 2020-05-24

**Authors:** Yue Yang, Xiaoxiong Liu, Weiguo Zhang, Xuhang Liu, Yicong Guo

**Affiliations:** 1 School of Automation, Northwestern Polytechnical University, Xi’an 710129, China; yangyue@mail.nwpu.edu.cn (Y.Y.); zhangwg@nwpu.edu.cn (W.Z.); liuxuhang@mail.nwpu.edu.cn (X.L.); guoyicong@mail.nwpu.edu.cn (Y.G.); 2 Key Laboratory of Flight Control and Simulation Technology, Northwestern Polytechnical University, Xi’an 710129, China

**Keywords:** nonlinear double model, federated EKF, multisensor model, small UAV, state estimation

## Abstract

Aimed at improving upon the disadvantages of the single centralized Kalman filter for integrated navigation, including its fragile robustness and low solution accuracy, a nonlinear double model based on the improved decentralized federated extended Kalman filter (EKF) for integrated navigation is proposed. The multisensor error model is established and simplified in this paper according to the near-ground short distance navigation applications of small unmanned aerial vehicles (UAVs). In order to overcome the centralized Kalman filter that is used in the linear Gaussian system, the improved federated EKF is designed for multisensor-integrated navigation. Subsequently, because of the navigation requirements of UAVs, especially for the attitude solution accuracy, this paper presents a nonlinear double model that consists of the nonlinear attitude heading reference system (AHRS) model and nonlinear strapdown inertial navigation system (SINS)/GPS-integrated navigation model. Moreover, the common state parameters of the nonlinear double model are optimized by the federated filter to obtain a better attitude. The proposed algorithm is compared with multisensor complementary filtering (MSCF) and multisensor EKF (MSEKF) using collected flight sensors data. The simulation and experimental tests demonstrate that the proposed algorithm has a good robustness and state estimation solution accuracy.

## 1. Introduction

Unmanned aerial vehicles (UAV) have taken a prominent role in a variety of applications, mostly military but also civilian. There are many significant advantages to using UAVs, such as for aerial surveillance [1], three-dimensional mapping models [2], search and rescue [3], and inspection [4] in complex environments. In recent years, there has been great interest in developing autonomous unmanned flight systems (AUFS) with advanced onboard capabilities, which include three critical types of technology: those of navigation, guidance, and control. The main focus of this paper is the research of navigation algorithm technology [5]. Traditionally, navigation [6] is the process of monitoring and controlling the UAV from one place to another place. In the broad sense, it can be defined as the process of data acquisition, analysis, extraction, and the inference of information. Moreover, it is a vital part of the UAV to interact with complex environments, and provide efficient and reliable state estimation parameters, which include attitude, velocity, and position. The accurate state estimation can help the UAV to perform trajectory control using certain advanced control system methods, such as the P-PI cascade control system [7], and the artificial cognitive control system based on a reinforcement learning strategy [8]. In particular, the accuracy of the sensors is limited because of the small UAV hardware performance and carrier space, but the multisensor Kalman filter fusion algorithm [9] allows us to construct state estimation from the different sensors. Moreover, progress in electronics miniaturization and microprocessors [10,11] has made it possible to house these sensors in small and compact devices. In addition, with the rapid development of MEMS [12], the cost of navigation devices has reduced, which brings the field of multisensor fusion algorithms into a period of rapid growth.

In the research of UAV navigation technology, the inertial navigation system (INS) and global satellite navigation system (GNSS) are indispensable. The INS [13,14], which depends on the inertial measurement unit (IMU), can provide the attitude, velocity, and position. Although INS has functional autonomy and a good reliability, and is not disturbed by the external environment, it has the disadvantage that the accumulation of INS errors can increase during the dead reckoning process, especially for low-cost IMUs. Thus, other navigation systems, e.g., global satellite navigation system (GNSS) [14,15], vision navigation [16], lidar simultaneous localization and mapping (Lidar-SLAM) [17], etc., can be employed to assist the INS with long-term precision. The GNSS can provide the velocity and position through the GNSS receiver [18] without signal shadows. Almost all outdoor UAVs are equipped with the GNSS receiver to provide their absolute location [19], and the position error of GNSS is about 1–2 m in the open field. Therefore, for a single navigation system with low-cost devices, the navigation data accuracy makes it difficult to meet the flight needs. A multisensor-integrated navigation system approach, which utilizes the complementarity of different navigation systems, can solve the issues of single navigation systems.

Nowadays, the Kalman filter [20,21], which is considered to be an optimal filter for the linear system model and statistical properties of Gaussian white noise, is widely used in many fields. In the multisensor-integrated navigation system, multiple navigation systems can be fused with the Kalman filter to obtain state estimation parameters. As a result of the UAV in-flight maneuverability and the complexity of the flight environment, the multisensor-integrated navigation model has the characteristics of nonlinearity and uncertainty. However, the Kalman filter, which is only applicable to linear Gaussian systems, is no longer optimal. Thus, a few improved nonlinear filter algorithms [22] have been proposed by certain scholars, such as the extended Kalman filter (EKF) [23], the unscented Kalman filter (UKF) [24,25], cubature Kalman filter (CKF) [26], particle filter (PF) [27], etc. In order to solve the parameter divergence caused by the strapdown inertial navigation system (SINS)/GPS model error, a quadratic EKF algorithm was proposed by Wang and Liu [28]. It can reduce the calculation by retaining the second-order derivation information and two-stage cascade estimation. In addition, for the low accuracy in filtering resulting from motion model errors, Hu and Wang [29] proposed a method based on the establishment of INS/GPS-integrated navigation by the UKF, which embeds the suboptimal fading factor (SFF) in the prediction covariance. Furthermore, a refined strong tracking UKF (RSTUKF) was developed to increase the robustness of UKF-resistant motion model errors. Liu and Qu [30] proposed a method combining the maximum correntropy criterion (MCC) and square-root cubature Kalman filter (SCKF) to apply to the SINS/GPS-integrated system, which not only retains the advantages of SRCF, but also exhibits a robust performance against heavy-tailed non-Gaussian noise.

Everything described above refers to centralized nonlinear navigation filters, which have several drawbacks. These include the heavy computational burden, the high state dimension, and the uncertain filter divergence. Compared with centralized filters, decentralized filters [31] use the redundancy information to detect and isolate the fault system, and construct the remaining subsystems, which can continue to complete the required task. Carlson [32,33] proposed a decentralized filter approach named the federated filter, which is being widely used because of its flexible design, small calculation costs, and good fault tolerance. When one or several navigation subsystems fail, it is easy to detect and separate the faults [34], and the remaining normal navigation subsystems can be quickly constructed to give the required filter states. Furthermore, it is reliable for the fusion algorithm from the local filter to global filter. For the navigation error model, Liang and Jia [35] proposed a fusion framework based on the federated filter, which is composed of EKF and differential decoupling KF (DDKF) for fault tolerance. Meanwhile, the prediction covariance matrix is extended by using the fault factor. Finally, the fault state and measurement are processed normally. Therefore, the federated filter [33] is very suitable for multisensor-integrated navigation as a new decentralized filter approach. It is often used for information distribution, as it is allocated among the different subsystems, and helps to improve filter performance.

In this paper, with a focus on model nonlinearity and state solution accuracy, a nonlinear double-model multisensor-integrated navigation method is proposed based on the federated extended Kalman filter fusion framework. A nonlinear double-model system consists of the nonlinear attitude heading reference system (AHRS) model and the nonlinear SINS/GPS model. The nonlinear attitude heading reference system model was used to perform the first local EKF sensor fusion to estimate the attitude error $\varphi^{n,1}$ and gyroscope bias $\varepsilon_{r}^{b,1}$ in Equation (22). The nonlinear SINS/GPS-integrated navigation model was used to perform the second local EKF sensor fusion to estimate the attitude error $\delta \varphi^{n,2}$, velocity error $\varepsilon_{r}^{b,2}$, position error $\delta v^{n,2}$, gyroscope bias $\delta p^{n,2}$, and accelerometer bias ${▽}_{r}^{b,2}$ in Equation (23). Then, the common states ($\delta \varphi^{n}$ and $\varepsilon_{r}^{b}$ in Equation (24)) of the nonlinear double model were optimized by the main filter to obtain the best attitude estimation. The main contributions of this study relative to other studies include the following:

1. The multisensor error mathematical model, which includes the gyroscope error model, the accelerometer error model, and the SINS error model, are described in this paper. Furthermore, these error models are reasonably simplified to apply to small-UAV near-ground short distance navigation;

2. A federated EKF approach is proposed combined with the federated filter and EKF, which not only solves the model nonlinearity, but also retains excellent filter precision;

3. During UAV flight, the requirement of attitude solution accuracy is of great importance. Thus, a nonlinear double model is proposed, including the nonlinear AHRS model and the nonlinear SINS/GPS model. The common attitude solutions of the nonlinear double model are fused in the main filter of the federated filter.

The rest of this paper is organized as follows. In Section 2, the multisensor error mathematical model is introduced and simplified. The double-model EKF algorithm for the federated filter is proposed in Section 3. Then, the simulation and experimental test in Section 4 demonstrate the performance of the proposed algorithm. Finally, the conclusions are given in Section 5.

## 2. Multisensor Error Mathematical Model

### 2.1. Gyroscope Error Model

The zero offset bias is the main influence factor of the gyroscope output precision when the UAV is in a static situation. Therefore, eliminating the zero offset error can improve the angular velocity accuracy of the gyroscope measurement. However, the random drift error of the gyroscope will change when the UAV is in flight, so the gyroscope error needs to be modeled [36] and estimated.

(1) $$\varepsilon_{g}=\varepsilon_{0}+\varepsilon_{r}+\omega_{g},$$

where $\varepsilon_{0}$ is the gyroscope constant error, $\varepsilon_{r}$ is the gyroscope random drift error, and $\omega_{g}$ is the gyroscope error white noise. The first-order Markov model of the gyroscope random drift error $\varepsilon_{r}$ is Equation (2).

(2) $${\dot{\varepsilon }}_{r}=-\frac{1}{\tau_{g}}\varepsilon_{r}+\omega_{gr},$$

where $\tau_{g}$ is the first-order Markov time constant, and $\omega_{gr}$ is the first-order Markov random white noise. Actually, $\varepsilon_{0}$ is the constant when the gyroscope is running, and it can be derived by Equation (3).

(3) $$\left\{\begin{array}{c} {\dot{\varepsilon }}_{0}=0\\ {\dot{\varepsilon }}_{0}+{\dot{\varepsilon }}_{r}={\dot{\varepsilon }}_{r} \end{array}\right.$$

Therefore, the constant error can be taken into account in the random drift error. The gyroscope error model [37] can be derived from Equations (1)–(3).

(4) $$\varepsilon_{g}=\varepsilon_{r}+\omega_{g}$$

### 2.2. Accelerometer Error Model

Typically, the error of the accelerometer includes the constant error and random drift error. Constant error can be eliminated using sensor calibration [38]. However, random drift error is time-varying and can be estimated using the filter algorithm to compensate. The accelerometer error model [35] can be represented by Equation (5).

(5) $${▽}_{a}={▽}_{0}+{▽}_{r}+\omega_{a},$$

where ${▽}_{0}$ is the constant error, ${▽}_{r}$ is the random drift error, and $\omega_{a}$ is the white noise of the accelerometer. The random drift error can be assumed to be the first-order Markov time constant in this paper, and can be derived from Equation (6).

(6) $${\dot{▽}}_{r}=-\frac{1}{\tau_{a}}{▽}_{r}+\omega_{ar},$$

where $\tau_{a}$ is the first-order Markov time constant, and $\omega_{ar}$ is the white noise. The constant error can be taken into account in the random drift error, the accelerometer error model can be derived from Equations (5) and (6).

(7) $${▽}_{a}={▽}_{r}+\omega_{a}$$

### 2.3. SINS Error Model

The attitude of the SINS error model [13] can be written as Equation (8).

(8) $$\delta \dot{\varphi }=\delta \varphi \times \omega_{in}^{n}+\delta \omega_{in}^{n}-\varepsilon_{g}^{n},$$

where δφ is the attitude error of the UAV, $\varepsilon_{g}^{n}$ is gyroscope bias in the navigation system, $\omega_{in}^{n}$ is the angular rate in the navigation system relative to the inertial system, and $\varepsilon_{g}^{n}$ is the gyroscope error in the navigation system.

The velocity of the SINS error model [13] can be represented by Equation (9).

(9) $$\delta {\dot{v}}^{n}=-\delta \varphi \times f^{n}+\delta v^{n}\times \left(2\omega_{ie}^{n}+\omega_{en}^{n}\right)+v^{n}\times \left(2\delta \omega_{ie}^{n}+\delta \omega_{en}^{n}\right)+{▽}_{a}^{n},$$

where $v^{n}$ is the velocity of the UAV, $\delta v^{n}$ is the velocity error in the navigation system, φ is the attitude of the UAV, $f^{n}$ is the special force of the accelerometer, $\omega_{ie}^{n}$ is the angular rate in the earth system relative to the inertial system, $\omega_{en}^{n}$ is angular rate in the navigation system relative to the earth system, and ${▽}_{a}^{n}$ is the accelerometer error in the navigation system.

The position of the SINS error model [13] can be described using Equation (10).

(10) $$\left\{\begin{array}{c} \delta \dot{L}=\frac{\delta v_{x}^{n}}{R_{e}+h}-\delta h\frac{v_{x}^{n}}{{\left(R_{e}+h\right)}^{2}}\\ \delta \dot{\lambda }=\frac{\delta v_{y}^{n}}{R_{e}+h}\sec L+\delta L\frac{v_{y}^{n}}{R_{e}+h}\tan L\sec L-\delta h\frac{v_{y}^{n}\sec L}{{\left(R_{e}+h\right)}^{2}}\\ \delta \dot{h}=-\delta v_{z}^{n}, \end{array}\right.$$

where ${\left[L\;\lambda \;h\right]}^{T}$ is the longitude, latitude, and height in the navigation system, respectively; ${\left[v_{x}^{n}\;v_{y}^{n}\;v_{z}^{n}\right]}^{T}$ is the velocity in the navigation system; and $R_{e}$ is the radius of earth.

In this paper, MEMS IMU sensors are used, which have a large noise compared to high-precision inertial devices. At the same time, the error state of the system only changes slightly under filter conditions, when the state of the SINS error equation selects the error quantity. Moreover, the SINS error model is quite complex in terms of computational complexity for the study of the embedded MEMS sensor fusion algorithm. Thus, there are some reasonable simplifications for the SINS error model in order to decrease the computational complexity and improve the update frequency of the navigation algorithm.

There is the pretty low height and relatively slow flight speed when a small UAV is in flight, and the update frequency of the proposed algorithm is high when the airborne CPU is running. Therefore, the rotation of navigation system caused by the change in the position of the UAV can be ignored during the algorithm running period. In addition, the measurement accuracy of the earth rotation angular rate is small relative to the gyroscope, and so the earth rotation angular rate is considered to be 0.

**Figure 1:**
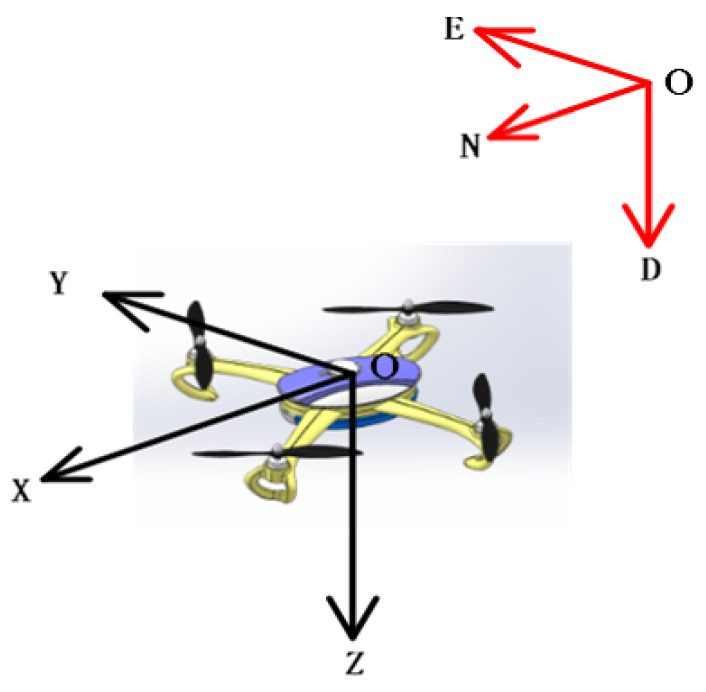
Unmanned aerial vehicle (UAV) coordinate system.

The UAV coordinate system is shown in Figure 1. In order to more intuitively observe the velocity and position change of the UAV, the navigation system is the north-east-down (N-E-D), and the body system is the front-right-down (X-Y-Z).

The nonlinear error model of Equations (8)–(10) is simplified, and the ${\left[L\;\lambda \;\;h\right]}^{T}$ is replaced by the $p^{n}={\left[p^{N}\;p^{E}\;p^{D}\right]}^{T}$ regarding the UAV position in the navigation system.

(11) $$\left\{\begin{array}{c} \delta {\dot{\varphi }}^{n}=-C_{b}^{n}\varepsilon_{r}^{b}-C_{b}^{n}\omega_{g}^{b}\\ \delta {\dot{v}}^{n}=C_{b}^{n}f^{b}\times \delta \varphi^{n}+C_{b}^{n}{▽}_{r}^{b}+C_{b}^{n}\omega_{a}^{b}\\ \delta {\dot{p}}^{n}=\delta v^{n}\\ {\dot{\varepsilon }}_{r}^{b}=-\frac{1}{\tau_{g}}\varepsilon_{r}^{b}+\omega_{gr}^{b}\\ {\dot{▽}}_{r}^{b}=-\frac{1}{\tau_{a}}{▽}_{r}^{b}+\omega_{ar}^{b} \end{array}\right.$$

where *b* is the body system, *n* is the navigation system, and $C_{b}^{n}$ is the direction cosine matrix from the body system to the navigation system.

## 3. The Double-Model EKF Algorithm of Federated Filter

### 3.1. Extended Kalman Filter

The Kalman filter problem generally is assumed to be linear in the mathematical model. However, the model in practical applications is a nonlinear system. In this paper, the designed method relies on the extended Kalman filter [23] for the nonlinear model system.

(12) $$\left\{\begin{array}{c} X_{k/k-1}=f\left(X_{k-1}\right)\\ P_{k/k-1}=\Phi_{k/k-1}P_{k-1}\Phi_{k/k-1}^{T}+Q_{k-1}\\ K_{k}=P_{k/k-1}H_{k}^{T}{\left(H_{k}P_{k/k-1}H_{k}^{T}+R_{k}\right)}^{-1}\\ X_{k}=X_{k/k-1}+K_{k}\left[Z_{k}-h\left(X_{k/k-1}\right)\right]\\ P_{k}=\left(I-K_{k}H_{k}\right)P_{k/k-1} \end{array}\right.$$

In Equation (12), the jacobian matrix of $\Phi_{k/k-1}$ and $H_{k}$ are obtained by the partial derivatives of $f(X_{k-1})$ and $h(X_{k/k-1})$, respectively.

(13) $$\left\{\begin{array}{c} \Phi_{k/k-1}=J\left(f\left(X_{k-1}\right)\right)\\ H_{k}=J\left(h\left(X_{k/k-1}\right)\right) \end{array}\right.$$

### 3.2. Federated Filter

There are generally two optimal combining methods for a multisensor-integrated navigation system: centralized KF and decentralized KF, respectively. Theoretically, centralized KF, which uses single KF to centrally process all the subsystem information of navigation, can give an optimal estimation of the state. As a result of the high state estimation dimension in practice, the computation is too large to run in real time for the algorithm. In addition, the centralized KF has a poor fault tolerance and is not conductive to fault diagnoses. However, the decentralized KF can use multiple filters to make estimations for the different subsystems of navigation so as to reduce error coupling. There are many decentralized KF algorithms currently. For example, an approach of decentralized algorithms named the federated filter was proposed by Carlson [32,33]. The federated filter is a special form of decentralized Kalman filtering method; it consists of several subsystem filters and a main filter. It is a decentralized filtering method with block estimation and a two-step cascade.

**Figure 2:**
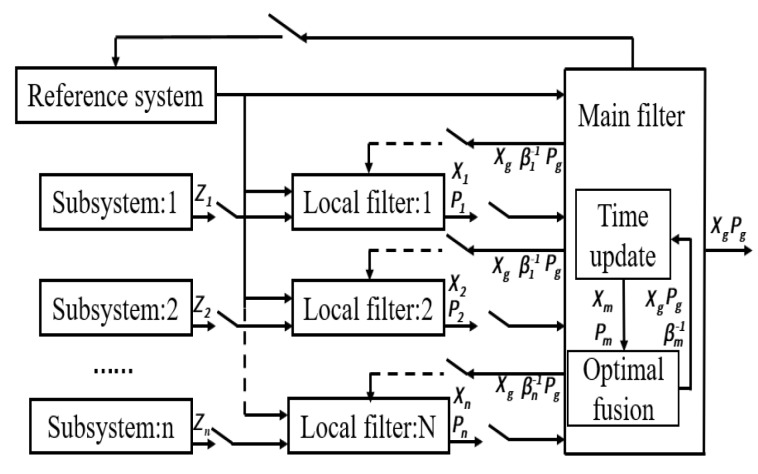
The block diagram of the federated filter.

The federated filter is often seen as a two-stage filter structure, as is shown in Figure 2. In this paper, the reference system in Figure 2 is the SINS; the output of which is given to the main filter on the one hand, and is also given to the local filter as the measurement value on the other. Then, the local state estimation and covariance are fed together with the main filter to obtain the global optimal estimation. $\beta_{i}\left(i=1,2,\ldots n\right)$ is the information distribution coefficient, and according to the principle of information distribution, different $\beta_{i}$ can be used to obtain the different structures and characteristics of the federated filter.

If there exists n local state estimations $X_{1},X_{2},\ldots ,X_{n}$ and the corresponding covariances $P_{1},P_{2},\ldots ,P_{n}$, ($P_{ij}=0\left(i\neq j\right)$), the global optimal estimation [39] can be expressed as Equation (14).

(14) $$\left\{\begin{array}{c} X_{g}=P_{g}\sum_{i=1}^{n}P_{i}^{-1}X_{i}\\ P_{g}={\left(\sum_{i=1}^{n}P_{i}^{-1}\right)}^{-1}, \end{array}\right.$$

where the $X_{g}$ and $P_{g}$ are the global estimation and covariance, respectively; and the $X_{i}$ and $P_{i}$ are the *i*th state estimation and covariance of the local filter, respectively.

(15) $$X_{i}=\left[\begin{array}{c} X_{ci}\\ X_{bi} \end{array}\right],$$

where the $X_{ci}$ is the common state estimation of the local filter, and $X_{bi}$ is the unique state estimation of the local filter. In this paper, we only fuse the common state estimation to get the global optimal estimation.

### 3.3. The Improved Federated EKF

The EKF has a good processing ability for nonlinear problems, and it is also easier to implement in practical applications. In addition, the federated filter has good fault tolerance compared to centralized KF. Moreover, the fault can be easily detected and isolated when the subsystem fails, then rest of the systems can be reconstructed. Thus, the proposed algorithm for filter precision is large and the computation of the fusion algorithm from the local filter to global filter is small, which is beneficial to execute in real time.

**Figure 3:**
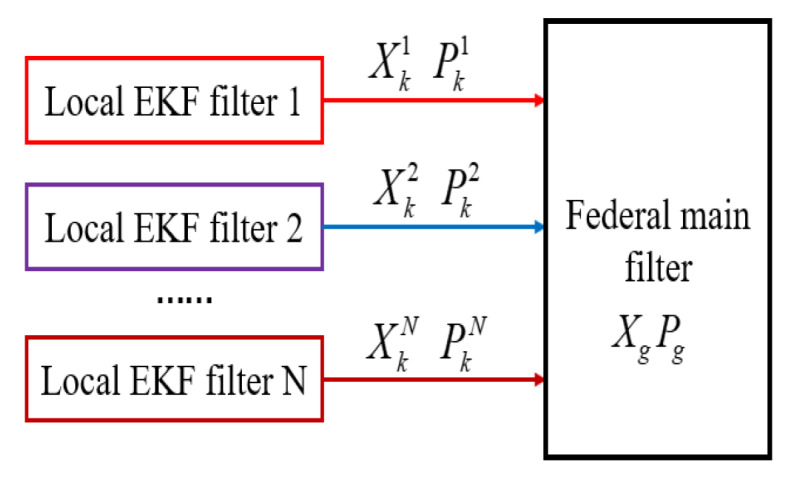
The improved federated EKF fusion diagram.

Aimed at the application of multisensor fusion, the improved federated EKF algorithm is proposed in this paper, which uses EKF to optimize the nonlinear model of the local filter, and then globally optimizes for the common state of the local EKF in order to meet the requirements of autonomous position navigation. Additionally, it can improve flight stability and reliability. Equation (16) is the improved federated EKF recursion formula.

(16) $$\left\{\begin{array}{c} X_{k/k-1}^{i}=f\left(X_{k-1}^{i}\right)\\ P_{k/k-1}^{i}=\Phi_{k/k-1}^{i}P_{k-1}^{i}{\left(\Phi_{k/k-1}^{i}\right)}^{T}+Q_{k-1}^{i}\\ K_{k}^{i}=P_{k/k-1}^{i}{\left(H_{k}^{i}\right)}^{T}{\left(H_{k}P_{k/k-1}^{i}{\left(H_{k}^{i}\right)}^{T}+R_{k}^{i}\right)}^{-1}\\ X_{k}^{i}=X_{k/k-1}^{i}+K_{k}^{i}\left[Z_{k}^{i}-h\left(X_{k/k-1}^{i}\right)\right]\\ P_{k}^{i}=\left(I-K_{k}^{i}H_{k}^{i}\right)P_{k/k-1}^{i}\\ X_{g}=P_{g}\sum_{i=1}^{n}{\left(P_{k}^{i}\right)}^{-1}X_{k}^{i}\\ P_{g}={\left(\sum_{i=1}^{n}{\left(P_{k}^{i}\right)}^{-1}\right)}^{-1} \end{array}\right.$$

where *i* is the *i*th local EKF, $X_{g}$ and $P_{g}$ are the results that are globally optimized for the common state estimation. The improved federated EKF fusion diagram is shown in Figure 3. Two local EKFs are used in this paper: one is to fuse the nonlinear AHRS model problem, and the other is to fuse the nonlinear SINS/GPS-integrated navigation problem, which will be introduced detail later.

### 3.4. The Nonlinear Double Model

Typically, this should be mathematically modeled when the actual system with nonlinear characteristics is described. Therefore, a unified nonlinear Gaussian state space model system is established for the multimodel of the federated filter based on the nonlinear theory [22].

(17) $$\left\{\begin{array}{c} X_{k}^{i}=f\left(X_{k-1}^{i}\right)+\omega_{k-1}^{i}\\ Z_{k}^{i}=h\left(X_{k}^{i}\right)+\nu_{k}^{i} \end{array}\right.$$

where *i* is the *i*th nonlinear model, $X_{k}^{i}$ is the state parameter, $f(X_{k-1}^{i})$ is the nonlinear state function, $\omega_{k-1}^{i}$ is the process noise, $Z_{k}^{i}$ is the measurement quantity, $h(X_{k}^{i})$ is the nonlinear measurement parameter, and $\nu_{k}^{i}$ is the measurement noise. Moreover, $\omega_{k-1}^{i}$ and $\nu_{k}^{i}$ are the Gaussian white noise.

#### 3.4.1. The Nonlinear AHRS Model

The AHRS can affect UAV flight stability, which is regulated by fusing the gyroscope, accelerometer, and magnetometer. According to the multisensor error mathematical model in Section 2, the nonlinear error model of AHRS in Equation (18) is established.

(18) $$\left\{\begin{array}{c} \delta {\dot{\varphi }}^{n,1}=-C_{b}^{n}\varepsilon_{r}^{b,1}-C_{b}^{n}\omega_{g}^{b,1}\\ {\dot{\varepsilon }}_{r}^{b,1}=-\frac{1}{\tau_{g}}\varepsilon_{r}^{b,1}+\omega_{gr}^{b,1} \end{array}\right.$$

where $\delta \varphi^{n,1}$ is the attitude error in the navigation system, $\omega_{g}^{b,1}$ is the Gaussian white noise of the gyroscope in the body system, and $\varepsilon_{r}^{b,1}$ is the bias of the gyroscope in the body system.

The measurement function of the AHRS can be written by Equation (19).

(19) $$Z_{k}^{1}=\left\{\begin{array}{c} \left[\begin{array}{c} \phi_{k}^{a}-{\hat{\phi }}_{k}\\ \theta_{k}^{a}-{\hat{\theta }}_{k} \end{array}\right]+\nu_{k}^{a}\\ \left(\psi_{k}^{m}-{\hat{\psi }}_{k}\right)+\nu_{k}^{b} \end{array}\right.$$

where $\phi_{k}^{a}$ and $\theta_{k}^{a}$ are the roll and pitch calculated by the accelerometer, respectively; $\psi_{k}^{m}$ is the yaw calculated by the magnetometer; ${\hat{\phi }}_{k}$, ${\hat{\theta }}_{k}$ and ${\hat{\psi }}_{k}$ are the attitude update value form the gyroscope; $\nu_{k}^{a}$ is the Gaussian white noise of the measurement from the accelerometer, and $\nu_{k}^{b}$ is the Gaussian white noise of the measurement from magnetometer.

#### 3.4.2. The Nonlinear SINS/GPS-Integrated Navigation Model

This paper uses the low-cost IMU that causes the state estimation to diverge over long running times. Thus, the GPS is selected as another subsystem of the multisensor-integrated navigation system of the UAV. The GPS, which has a good long-term stability, calculates the velocity and position of the carrier by utilizing the received satellite navigation signals. It can be seen that the SINS/GPS is the current mainstream choice because of the complementary performance of SINS and GPS. However, it is necessary to establish a nonlinear mathematical model that accurately reflects the system in order to better analyze the actual SINS/GPS system.

(20) $$\left\{\begin{array}{c} \delta {\dot{\varphi }}^{n,2}=-C_{b}^{n}\varepsilon_{r}^{b,2}-C_{b}^{n}\omega_{g}^{b,2}\\ {\dot{\varepsilon }}_{r}^{b,2}=-\frac{1}{\tau_{g}}\varepsilon_{r}^{b,2}+\omega_{gr}^{b,2}\\ \delta {\dot{v}}^{n,2}=C_{b}^{n}f^{b,2}\times \delta \varphi^{n,2}+C_{b}^{n}{▽}_{r}^{b,2}+C_{b}^{n}\omega_{a}^{b,2}\\ \delta {\dot{p}}^{n,2}=\delta v^{n,2}\\ {\dot{▽}}_{r}^{b,2}=-\frac{1}{\tau_{a}}{▽}_{r}^{b,2}+\omega_{ar}^{b,2} \end{array}\right.$$

where $\delta \varphi^{n,2}$, $\delta v^{n,2}$, and $\delta p^{n,2}$ are the errors for attitude, velocity, and position, respectively; $\varepsilon_{r}^{b,2}$ and ${▽}_{r}^{b,2}$ are the bias of the gyroscope and accelerometer, respectively; and $\omega_{gr}^{b,2}$ and $\omega_{ar}^{b,2}$ are the Gaussian white noise of the gyroscope and accelerometer, respectively.

The measurement function of the SINS/GPS can be established by Equation (21).

(21) $$Z_{k}^{2}=\left[\begin{array}{c} p_{k}^{a}-{\hat{p}}_{k}\\ v_{k}^{a}-{\hat{v}}_{k} \end{array}\right]+\nu_{k}^{c}$$

where $p_{k}^{a}$ and $v_{k}^{a}$ are the velocity and position of the GPS, respectively; ${\hat{p}}_{k}$ and ${\hat{v}}_{k}$ are the velocity and position of SINS, respectively; $\nu_{k}^{c}$ is the Gaussian white noise of the GPS.

### 3.5. Common State Fusion

The common state parameters of the nonlinear double model, which are the error of attitude $\delta \varphi^{n}$ and gyroscope bias $\varepsilon_{r}^{b}$, are fused by Equations (22) and (23) under the federated filter framework. In addition, the gyroscope bias and the accelerometer bias of the main filter are fed back into the SINS so as to reduce sensor errors.

(22) $$X^{A}=\left[\begin{array}{cc} \delta \varphi^{n,1} & \varepsilon_{r}^{b,1} \end{array}\right]$$

(23) $$X^{B}=\left[\begin{array}{ccccc} \delta \varphi^{n,2} & \varepsilon_{r}^{b,2} & \delta v^{n,2} & \delta p^{n,2} & {▽}_{r}^{b,2} \end{array}\right]$$

(24) $$X^{C}=\left[\begin{array}{c} \delta \varphi^{n}\\ \varepsilon_{r}^{b} \end{array}\right]$$

The error of attitude and the gyroscope bias of Equations (22) and (23) are taken as the common state estimation. On the one hand, the attitude is of a high accuracy because the UAV is a flexible, maneuverable aircraft; on the other hand, the accurate attitude can make the velocity and position of the UAV more reliable in the navigation system. The common state fusion algorithm of the federated filter is obtained from Equation (25).

(25) $$\left\{\begin{array}{c} X^{C}=P^{C}\left({\left(P^{A}\right)}^{-1}+{\left(P_{\left[1:6,1:6\right]}^{B}\right)}^{-1}X_{\left[1:6\right]}^{B}\right))\\ P^{C}={\left({\left(P^{A}\right)}^{-1}+{\left(P_{\left[1:6,1:6\right]}^{B}\right)}^{-1}\right)}^{-1} \end{array}\right.$$

where $X^{C}$ and $P^{C}$ are the common state estimation and covariance, respectively; $X^{A}$ and $P^{A}$ are the state estimation and covariance of the nonlinear AHRS model, respectively; $X^{B}$ and $P^{B}$ are the state estimation and covariance of the nonlinear SINS/GPS-integrated navigation model, respectively; $X_{\left[1:6\right]}^{B}$ is the error of attitude and gyroscope bias; and $P_{\left[1:6,1:6\right]}^{B}$ is the corresponding covariance.

The unique state parameter of the nonlinear SINS/GPS-integrated navigation model is used as part of the main filter state output in the federated filter framework.

(26) $$X=\left[\begin{array}{c} X^{C}\\ X_{\left[7:15\right]}^{B} \end{array}\right]$$

where $X_{\left[7:15\right]}^{B}$ is the unique state quantity which includes the velocity error, position error, and accelerometer bias.

### 3.6. The Federated Double-Model EKF Algorithm

The local EKF and federated filter were introduced in Section 2, and a federated EKF approach was proposed. After that, a nonlinear double model was designed in order to increase the solution accuracy and fault tolerance of the filter system. Finally, the federated double-model EKF multisensor fusion algorithm was investigated. The body system is “front-right-down”, and the navigation system is “north-east-down”.

**Figure 4:**
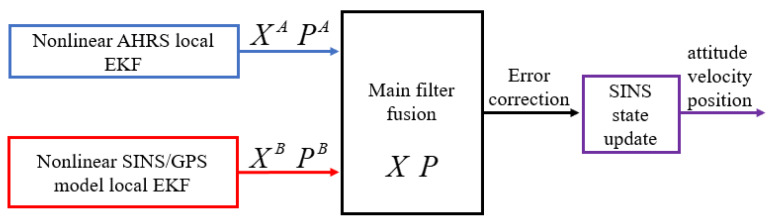
The federated DMEKF (Double Model EKF) fusion diagram.

The establishment of the federated double-model EKF algorithm can make state estimation more accurate, because the UAV requires higher accuracy for attitude calculations compared to ground robots. This problem is effectively solved by the nonlinear double model in this paper. The decentralized multimodel can globally optimize the common state (error of attitude and gyroscope bias) of the two nonlinear models, which is suitable for the rapid maneuvering of the UAV. The traditional SINS/GPS-integrated navigation diverges when the GPS signal is interfered with or invalid; however, the algorithm in this paper reconstructs the rest of the sensor information in the case of GPS failure, which can guarantee that the normal attitude is provided to the UAV. The federated double-model EKF fusion diagram is shown in Figure 4.

**Figure 5:**
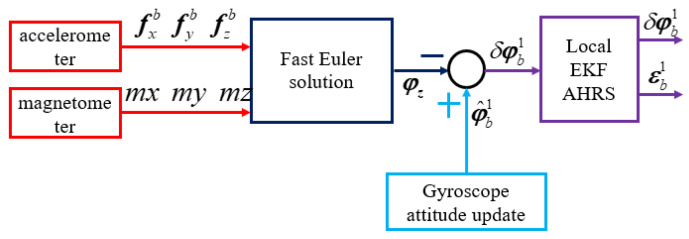
The nonlinear attitude heading reference system (AHRS) local extended Kalman filter (EKF) diagram.

The nonlinear AHRS local EKF system includes three kinds of sensors: gyroscope, accelerometer, and magnetometer. As shown in Figure 5, the measurement of attitude by the three-axis accelerometer and magnetometer is calculated by FastEuler solution, and subtracted from the attitude that is obtained by the gyroscope attitude update. Then, the local EKF AHRS fusion is used to optimize in order to obtain the error of attitude and gyroscope bias. The FastEuler solution is derived from Equations (27) and (28).

(27) $$\left\{\begin{array}{c} \phi_{a}=atan2\left(-f_{y}^{b},-f_{z}^{b}\right)\\ \theta_{a}=atan2\left(f_{x}^{b},-f_{z}^{b}\right) \end{array}\right.$$

(28) $$\left\{\begin{array}{c} hx=mx\cos\theta_{a}+my\sin\theta_{a}+mz\sin\theta_{a}\cos\phi_{a}\\ hy=my\cos\phi_{a}-mz\sin\phi_{a}\\ \psi_{m}=atan2\left(-hy,hx\right) \end{array}\right.$$

**Figure 6:**
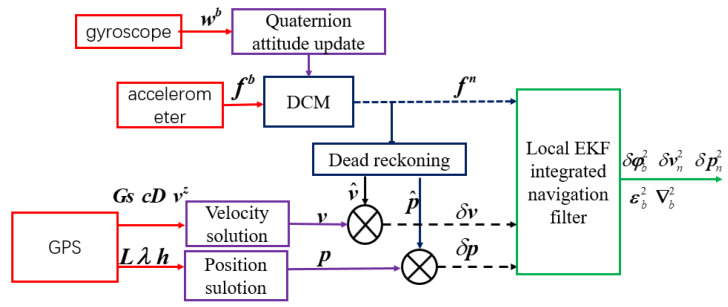
The nonlinear strapdown inertial navigation system (SINS)/GPS local EKF diagram.

The nonlinear SINS/GPS model system includes three kinds of sensors: the gyroscope, accelerometer, and GPS. As shown in Figure 6, the three-axis gyroscope angular velocity is calculated by the quaternion attitude update formula to obtain the direction cosine matrix $C_{b}^{n}$, and converting the three-axis accelerometer measurement from the body system to the navigation system in order to use the dead reckoning to calculate the velocity $\hat{v}$ and position $\hat{p}$. The longitude, latitude, and height of GPS data is calculated using Equation (29) to obtain the position p in the navigation system. The ground speed (Gndspd) and course degree (coursDeg) of the GPS data are solved using Equation (30) to obtain the velocity v in the navigation system. Then, the error measurement of the velocity and position are calculated using the $\left(\hat{v}-v\right)$ and $\left(\hat{p}-p\right)$. Finally, the local EKF is used to calculate the error of the attitude, velocity, and position, respectively.

(29) $$\left\{\begin{array}{c} p^{N}=R^{e}\Delta \lambda \backslash 100\\ p^{E}=R^{e}cos\left(L_{0}\frac{\pi }{180}\right)\Delta \lambda \backslash 100\\ p^{D}=\Delta h \end{array}\right.$$

(30) $$\left\{\begin{array}{c} v^{N}=Gndspd*cos\left(coureDed*\frac{\pi }{180}\right)\\ v^{E}=Gndspd*sin\left(coureDed*\frac{\pi }{180}\right),\\ v^{D}=v^{z} \end{array}\right.$$

where $R^{e}$ is the Earth’s radius (about 6,378,145 m), Δλ and Δh are the differences in latitude and height in the adjacent time interval, $L_{0}$ is the longitude at the initial time, the Gndspd and coursDed are the ground rate and course degree at the current time, respectively.

## 4. Simulation and Experimental Test

### 4.1. Data Acquisition and Flight Platform

**Figure 7:**
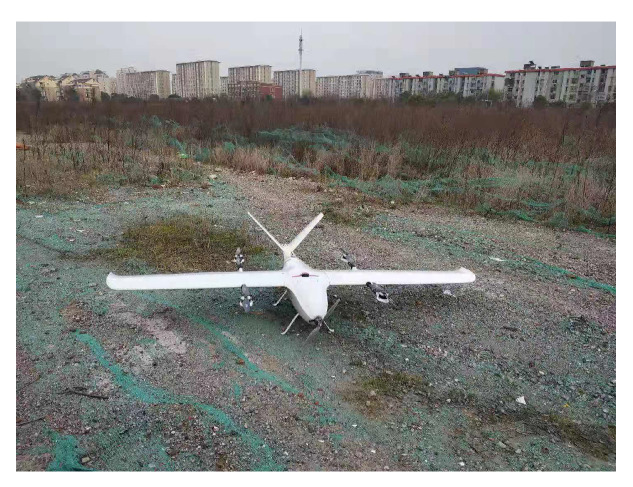
The experimental platform of the vertical take-off and landing (VTOL) UAV.

This paper used the experimental platform shown in Figure 7 to obtain the raw flight sensors data. It includes the three-axis angular velocity, three-axis acceleration, three-axis magnetic values, velocity of GPS, and position of GPS. The flight sensors data condition with GPS and without GPS were collected by the vertical take-off and landing (VTOL ) UAV.

### 4.2. Flight Sensors Data with GPS

The flight sensors data were collected with the GPS of the UAV using the experimental platform shown in Figure 7 whilst performing some maneuvers. The proposed federated double-model EKF (FDMEKF) was compared with multisensor complementary filtering (MSCF) (the MSCF takes advantage of the complementary nature of sensor frequencies to estimate the state parameters of the UAV) and the multisensor EKF (MSEKF) (the MSEKF is only used to process the nonlinear SINS/GPS model to estimate the state parameters of the UAV).

**Figure 8:**
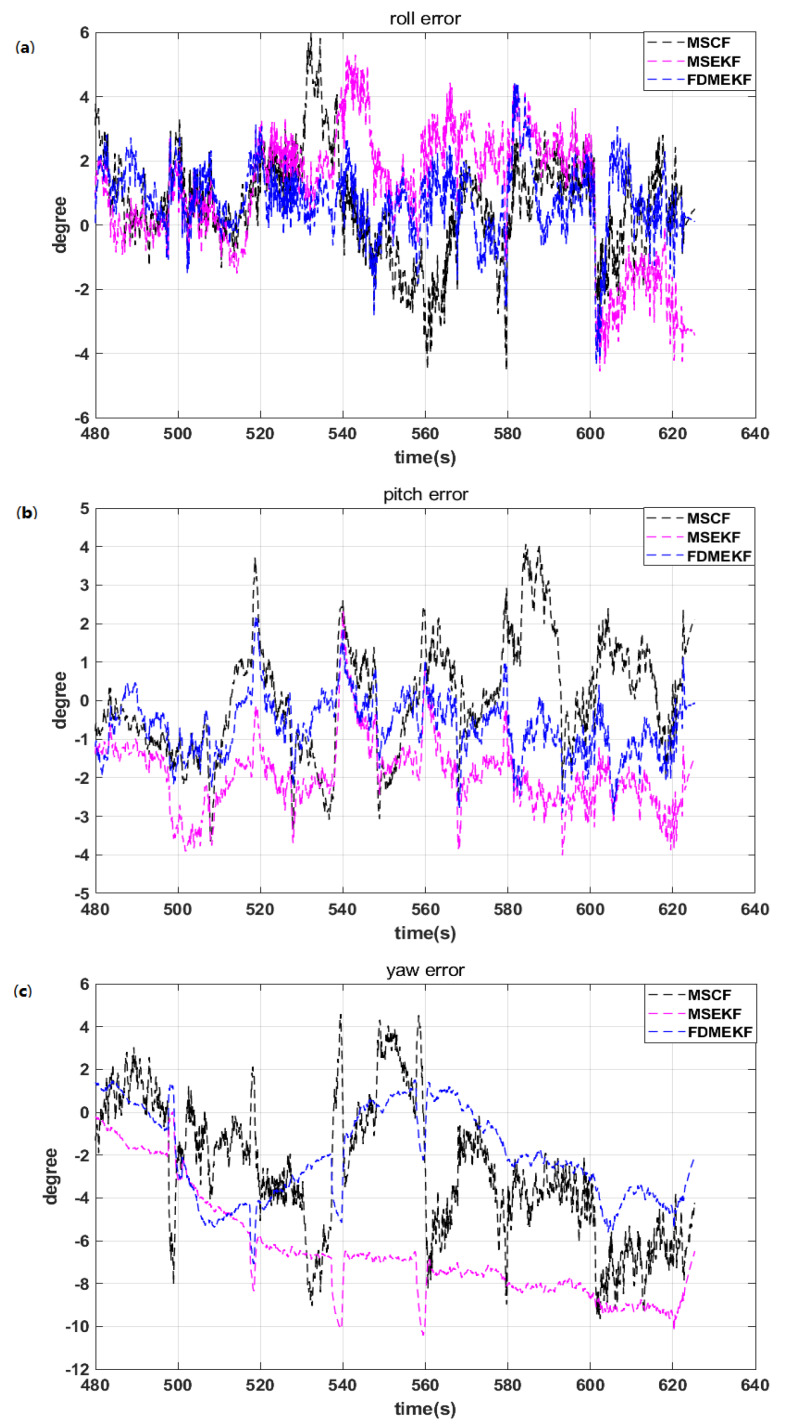
The attitude error of the UAV in flight. (**a**) the roll error of the UAV; (**b**) the pitch error of the UAV; (**c**) the yaw error of the UAV.

As can be seen in the previous figures, the attitude error of MSCF is the largest compared with the other two algorithms in Figure 8. The sensor noise is an important factor affecting the solution accuracy of the low-cost sensors. However, the MSCF cannot suppress the noise of the low-cost sensors very well. This is because the MSCF model does not consider the sensor noise, it just uses the complementary characteristics of the sensor frequency. The MSEKF model introduces the sensor noise in Equation (17) (i=1), and can handle the nonlinear SINS/GPS model. However, the state solution accuracy of the single nonlinear model is not as good as the FDMEKF. The FDMEKF not only considers the sensor noise in Equation (17) (i=2), but also describes the two local EKF methods to the nonlinear AHRS model and the nonlinear SINS/GPS model. Then, it fuses the common state of the two models to improve the attitude solution accuracy compared with the MSCF and the MSEKF.

**Figure 9:**
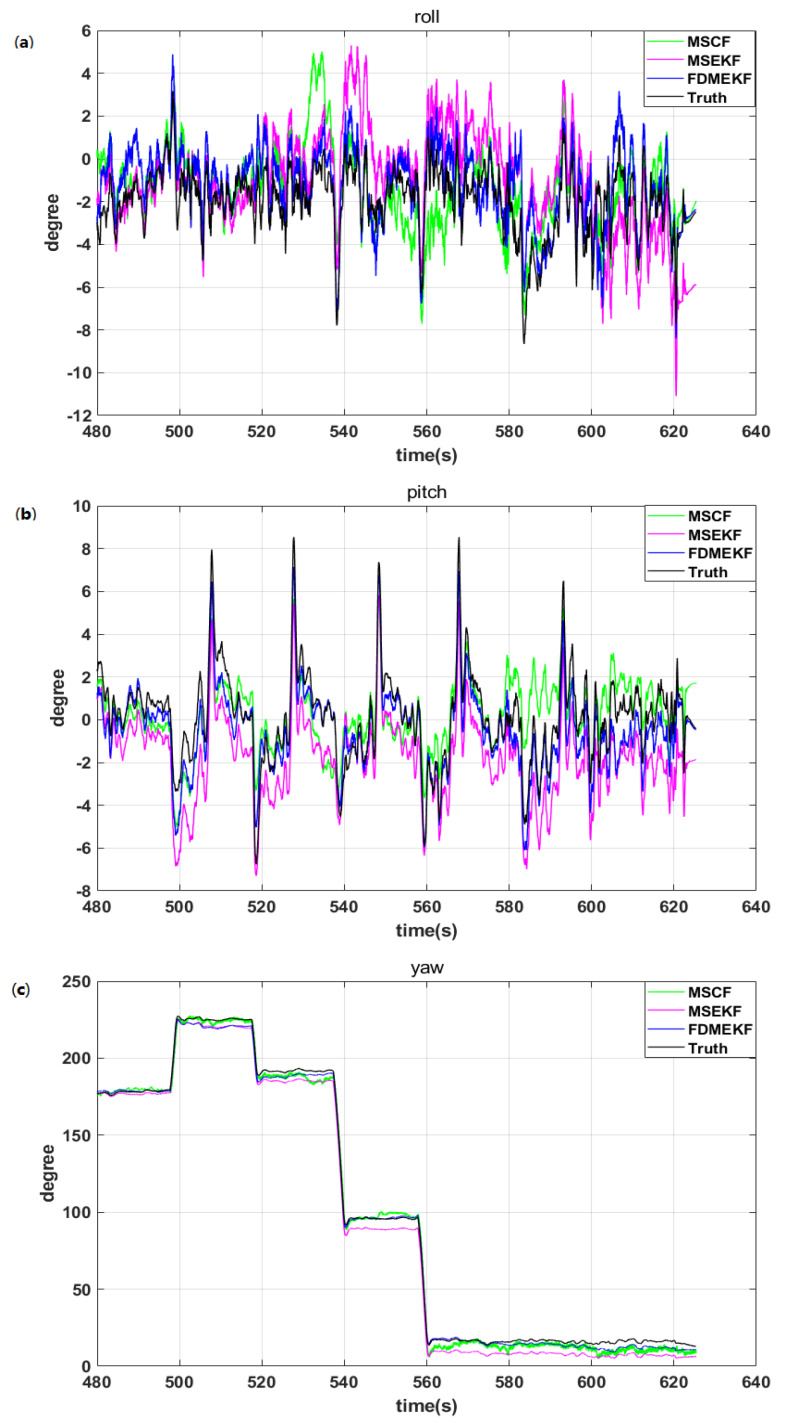
The attitude of the UAV in flight. (**a**) the roll of the UAV; (**b**) the pitch of the UAV; (**c**) the yaw of the UAV.

It can be seen that the attitude of MSCF is influenced by the sensor noise from the diagram of the UAV attitude in Figure 9, so that the roll of MSCF demonstrates an occasional abnormal burr phenomenon. The attitude of MSEKF has a certain deviation compared to the truth, and the roll of MSEKF does not track the roll of the truth after 540 s. Compared with the two previous kinds of filters, the attitude of the FDMEKF can track the truth very well. Firstly, since the FDMEKF can eliminate the sensor noise compared to the MSCF. Secondly, as a result of the nonlinear double model based on the decentralized filter, the filter accuracy is higher than the single model centralized filter, and is more suitable for the UAV. In order to more intuitively compare the attitude accuracy of the three filters, Table 1 provides the mean absolute errors (MAE), standard deviation (STD), and root mean square errors (RMSE) of the attitude obtained by MSCF, MSEKF, and FDMEKF. It can be seen that the MAE, STD, and RMSE of the FDMEKF are also much smaller than those of the other two filters. This is because the FDMEKF improves the attitude solution accuracy through fusing the common states of the two nonlinear models using Equation (14).

**Figure 10:**
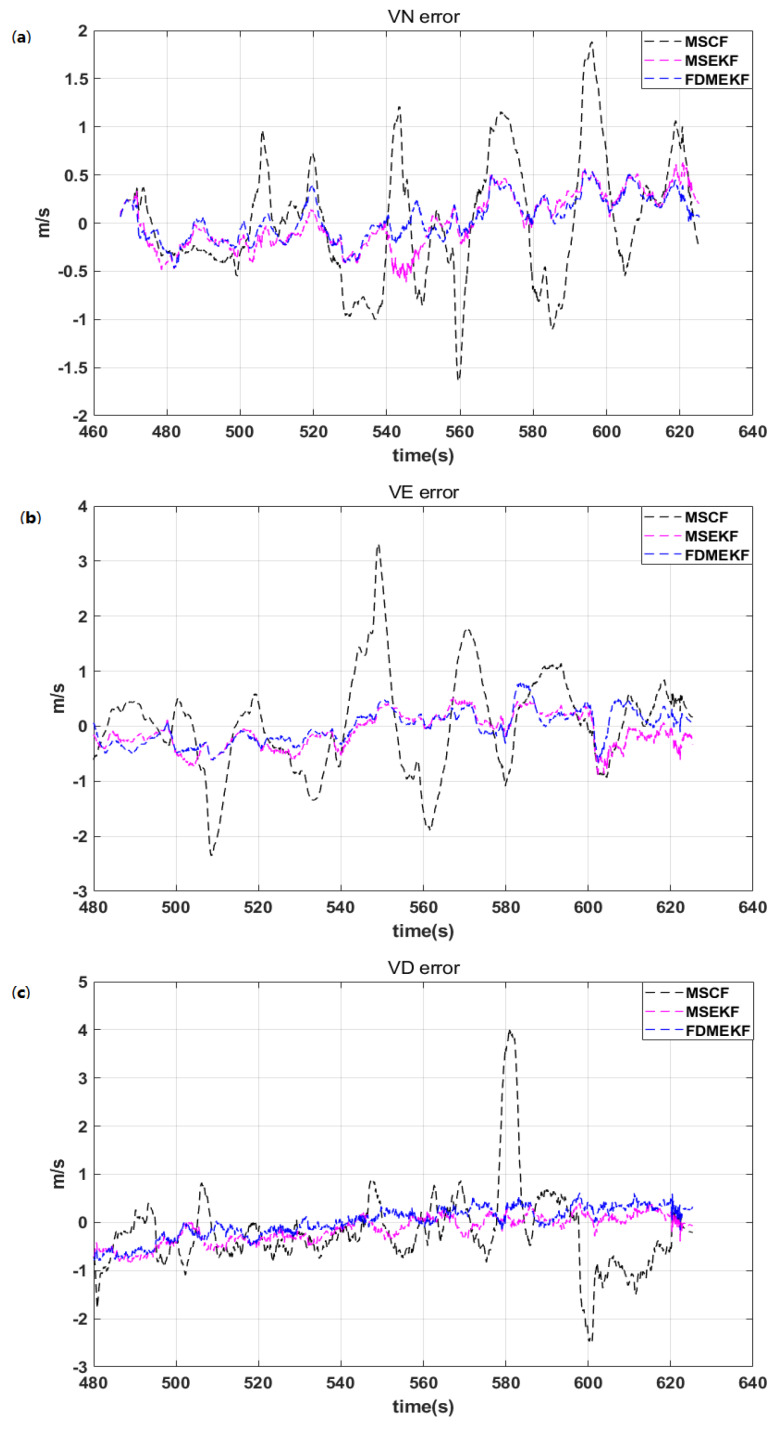
The velocity error the UAV in flight. (**a**) the north velocity error of the UAV; (**b**) the east velocity error of the UAV; (**c**) the down velocity error of the UAV.

Figure 10 depicts the velocity error of the UAV. It is evident that the velocity error of the FDMEKF is smallest because it considers the GPS velocity noise. Moreover, the improvement in the FDMEKF attitude accuracy reduces the cumulative error caused by the dead reckoning from acceleration.

**Figure 11:**
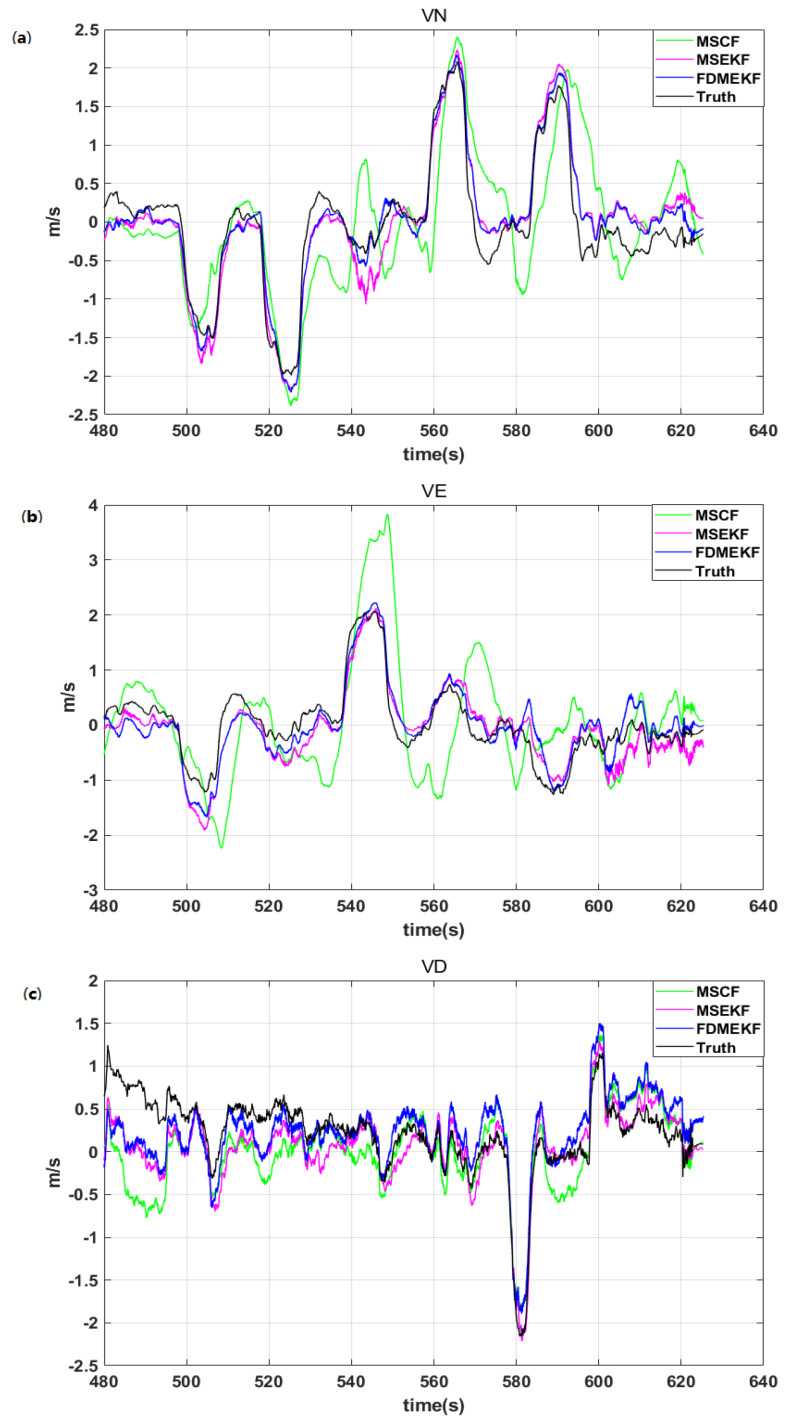
The velocity of the UAV in flight. (**a**) the north velocity of the UAV; (**b**) the east velocity of the UAV; (**c**) the down velocity of the UAV.

It can be seen that the FDMEKF can better track the velocity of the truth compared with the velocity of MSCF and the MSEKF in Figure 11, respectively. In the velocity of the MSCF, a large magnitude of oscillations appear in the filtering curve when the UAV performs the maneuver. This is because the MSCF does not eliminate the large GPS velocity noise from the low-cost sensors. Table 2 provides the mean absolute errors (MAE), standard deviations (STD), and root mean square errors (RMSE) of the velocity obtained by MSCF, MSEKF, and FDMEKF. It can be clearly seen that the FDMEKF has a good performance in terms of the UAV velocity solution accuracy.

**Figure 12:**
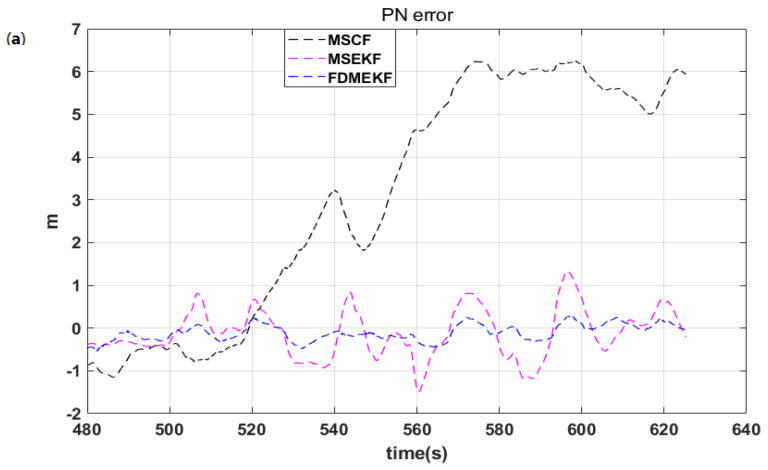

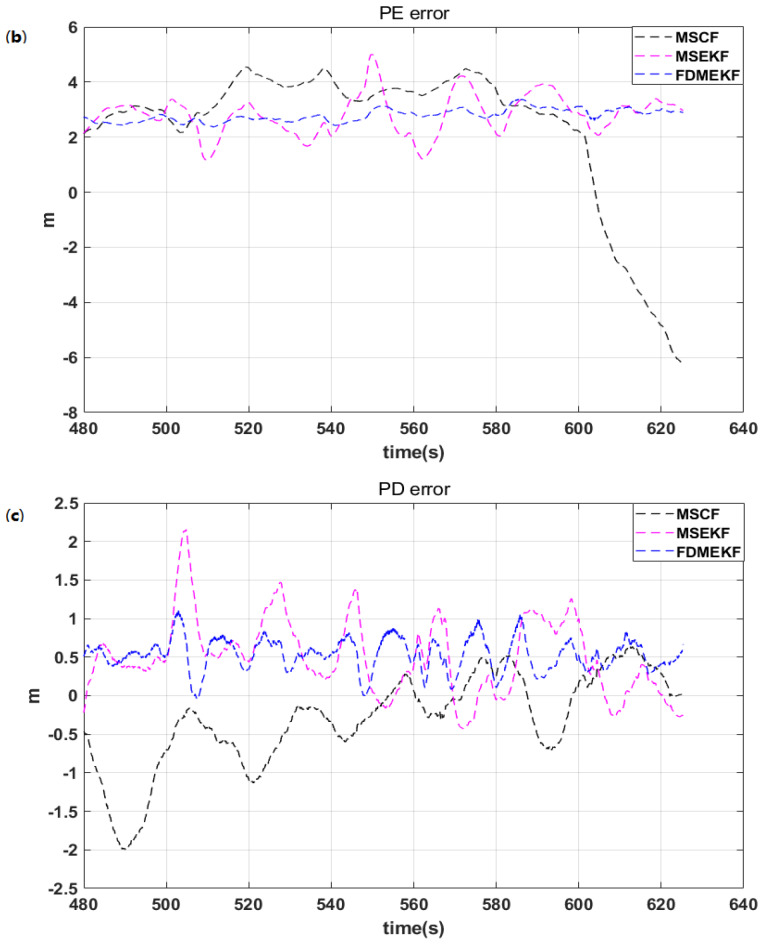
The position error of the UAV in flight. (**a**) the north position error of the UAV; (**b**) the east position error of the UAV; (**c**) the down position error of the UAV.

Figure 12 shows the position error of the UAV. It is evident that the position error of MSCF is large because it ignores the GPS position noise when calculating the position solution. Meanwhile, it was already shown that the velocity error of the FDMEKF is the smallest. When the velocity is integrated to get the position, the velocity error is transferred to the position error. Thus, the velocity error also affects the position error. Therefore, the position error of the FDMEKF is also better than the other two filters.

**Figure 13:**
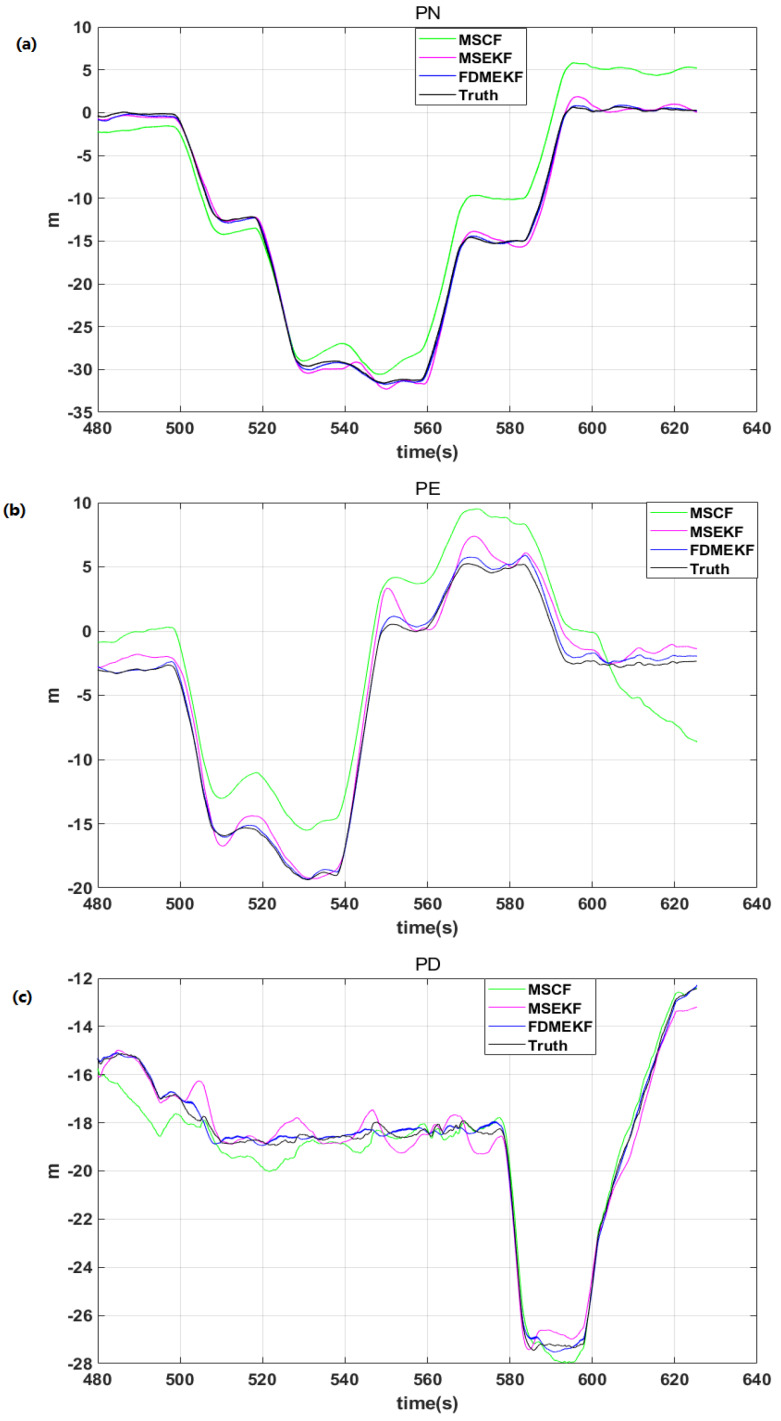
The position of the UAV in flight. (**a**) the north position of the UAV; (**b**) the east position of the UAV; (**c**) the down position of the UAV.

It can be found that the FDMEKF can better track the position of truth compared with the position of MSCF and the MSEKF in Figure 13, respectively. Moreover, the position curves of the FDMEKF are smoother than those of the MSCF and the MSEKF. Table 3 provides the mean absolute errors (MAE), standard deviations (STD), and root mean square errors (RMSE) of the position obtained by MSCF, MSEKF, and FDMEKF. It illustrates that the position solution accuracy of the FDMEKF is better than the MSCF and the MSEKF.

### 4.3. Flight Sensors Data without GPS

The flight sensors data without GPS of the UAV were collected by the flight platform as shown in Figure 7. This is to verify that when the GPS fails, the proposed algorithm can still provide a reliable attitude for the UAV.

**Figure 14:**
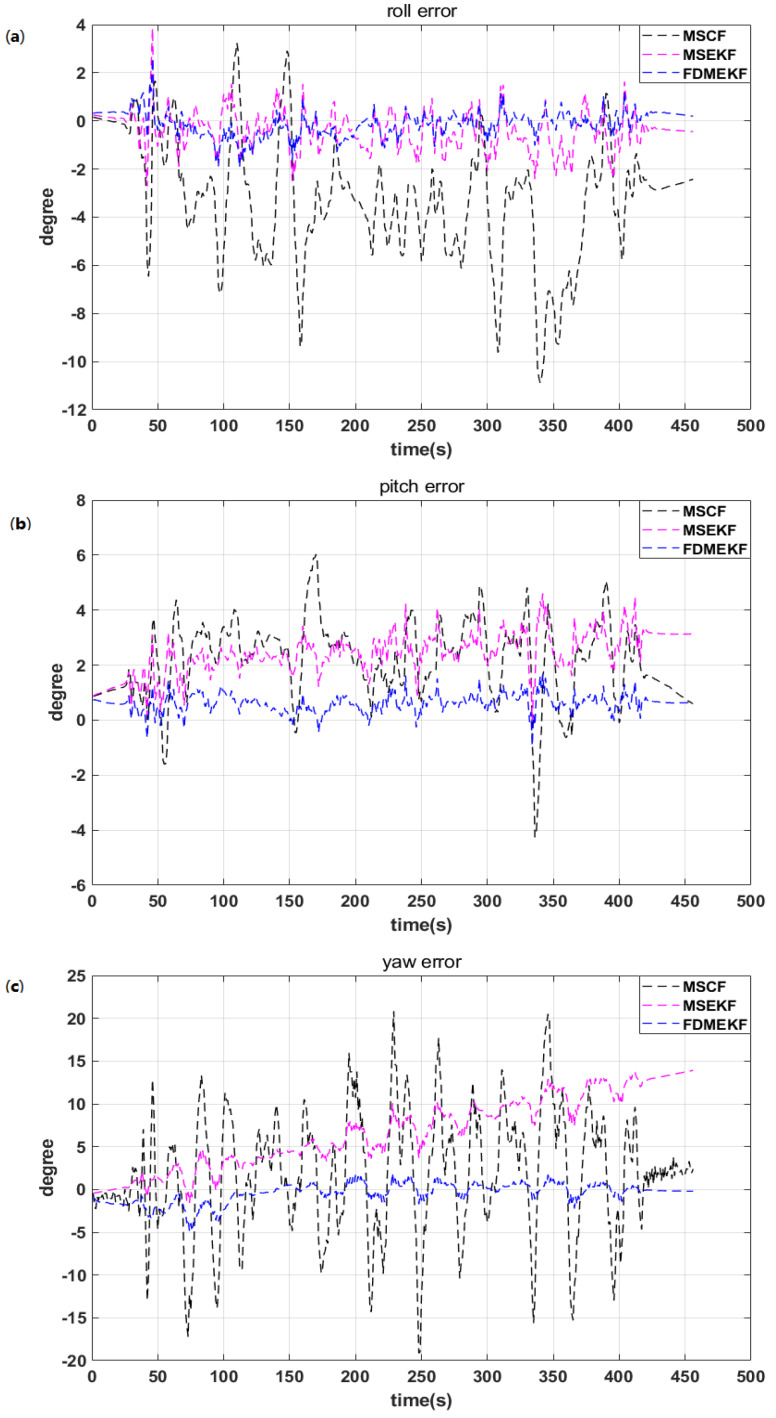
The attitude error of the UAV in flight. (**a**) the roll error of the UAV; (**b**) the pitch error of the UAV; (**c**) the yaw error of the UAV.

The velocity and position of the state estimation parameters are inaccurate when GPS signal is disturbed in city buildings and jungle environment. At this time, the reliable attitude solution is very important when the UAV is in flight. Figure 14 shows that the attitude error curves of the MSCF and the MSEKF have large fluctuations. The attitude error of the FDMEKF can converge to 2 degrees. The nonlinear SINS/GPS of the double model fails when the GPS signal is disturbed, but the FDMEKF can still provide the reliable attitude depending on the nonlinear AHRS of the double model.

**Figure 15:**
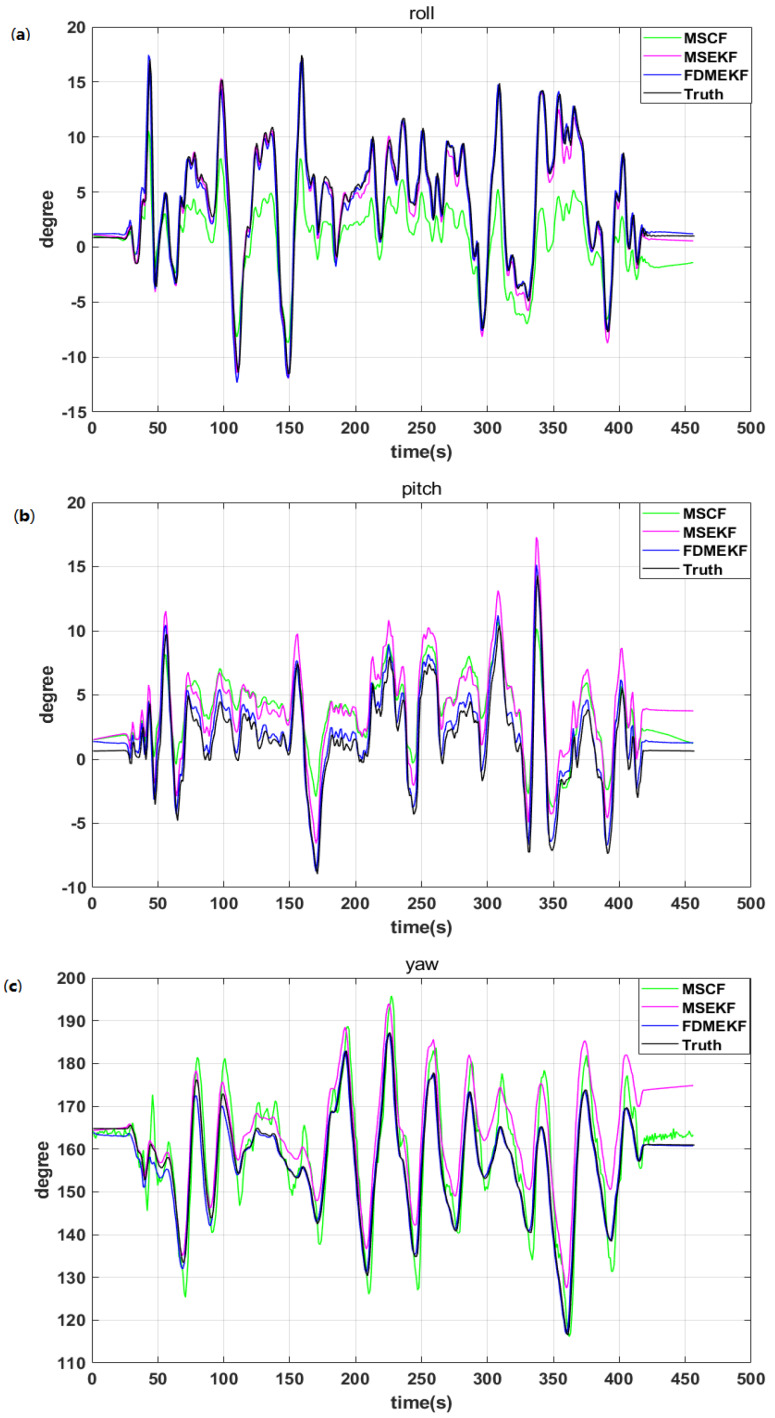
The attitude the UAV in flight. (**a**) the roll of the UAV; (**b**) the pitch of the UAV; (**c**) the yaw of the UAV.

It can be seen that the attitude curves of the FDMEKF are closest to the truth compared with the MSCF and MSEKF, as shown in Figure 15. The attitude solution of the MSCF is inaccurate due to the lack of the low frequency characteristics of the GPS signal. Meanwhile, the attitude solution of the MSCF has a divergent trend due to the failure of the nonlinear SINS/GPS model. Table 4 provides the mean absolute errors (MAE), standard deviations (STD), and root mean square errors (RMSE) of the attitude obtained by the MSCF and MSEKF. It clearly illustrates that the FDMEKF can still provide a good attitude solution accuracy when the GPS signal fails.

## 5. Conclusions

A nonlinear double model approach based on the decentralized federated extended Kalman filter (EKF) is proposed in this paper for the multisensor fusion algorithm of small UAVs. The contributions of this paper include the following: (1) The multisensor error mathematical model is established and simplified to a reasonable extent; (2) the federated EKF is further developed to enhance the filter accuracy and robustness; (3) a novel nonlinear double model is designed to obtain a more accurate attitude solution for cases in which there is GPS and there is not. Simulations and experimental test results demonstrate that the proposed filter algorithm can effectively provide the state estimation in order to meet the flight requirements of a UAV, as well as having a much higher solution accuracy.

## Figures and Tables

**Table 1:** Mean absolute errors (MAE), standard deviations (STD), and root mean square errors (RMSE) of the attitude achieved by multisensor complementary filtering (MSCF), multisensor EKF (MSEKF), and the federated double-model EKF (FDMEKF) for the experimental case.

Methods		Attitude (deg)		
		Roll	Pitch	Yaw
MSCF	MAE	1.702	1.985	8.065
	STD	2.183	1.706	8.560
	RMSE	1.822	1.434	3.984
MSEKF	MAE	1.651	1.824	8.008
	STD	2.032	1.673	8.260
	RMSE	1.437	1.265	3.460
FDMEKF	MAE	1.564	1.528	7.897
	STD	1.921	1.504	8.417
	RMSE	1.336	1.022	2.817

**Table 2:** MAE, STD, and RMSE of the velocity achieved by MSCF, MSEKF, and FDMEKF for the experimental case.

Methods		Velocity (m/s)		
		VN	VE	VD
MSCF	MAE	0.633	0.786	0.398
	STD	0.879	1.093	0.535
	RMSE	0.641	0.907	0.695
MSEKF	MAE	0.534	0.465	0.325
	STD	0.859	0.829	0.495
	RMSE	0.576	0.716	0.585
FDMEKF	MAE	0.492	0.435	0.309
	STD	0.572	0.529	0.246
	RMSE	0.314	0.476	0.447

**Table 3:** MAE, STD, and RMSE of the position achieved by MSCF, MSEKF, and FDMEKF for the experimental case.

Methods		Position (m)		
		PN	PE	PD
MSCF	MAE	0.940	0.836	1.209
	STD	1.879	1.103	1.535
	RMSE	0.566	0.873	0.672
MSEKF	MAE	0.724	0.617	0.876
	STD	0.959	0.729	0.995
	RMSE	0.361	0.702	0.581
FDMEKF	MAE	0.692	0.531	0.601
	STD	0.658	0.503	0.691
	RMSE	0.223	0.523	0.512

**Table 4:** MAE, STD, and RMSE of the attitude achieved by MSCF, MSEKF, and FDMEKF for the experimental case.

Methods		Attitude (deg)		
		Roll	Pitch	Yaw
MSCF	MAE	3.698	2.739	11.135
	STD	4.733	3.449	14.197
	RMSE	4.097	2.557	7.340
MSEKF	MAE	4.133	3.300	11.148
	STD	5.265	4.235	13.761
	RMSE	0.885	2.549	7.982
FDMEKF	MAE	0.970	0.452	1.565
	STD	2.249	1.508	2.495
	RMSE	0.543	0.689	1.231
